# A tripartite biopsychosocial intervention program enhances mental health and quality of life in elderly patients with depression secondary to geriatric diseases

**DOI:** 10.3389/fpsyg.2025.1696674

**Published:** 2025-10-06

**Authors:** Yan Chen, Xiao Lin, Xinwu Ye

**Affiliations:** ^1^Department of Geriatric Psychiatry, Wenzhou Seventh People's Hospital, Wenzhou, Zhejiang, China; ^2^Department of Psychosomatic Medicine, Wenzhou Seventh People's Hospital, Wenzhou, Zhejiang, China

**Keywords:** geriatric disease, secondary depression, trinity of physical, psychological, and social functions, mental health, quality of life

## Abstract

**Objective:**

To evaluate the effectiveness of a tripartite biopsychosocial intervention program on mental health and quality of life in elderly patients with depression secondary to geriatric diseases.

**Methods:**

A cluster randomized controlled trial was conducted with 166 elderly patients between January 2022 and January 2025. Wards were randomized into an intervention group (*n* = 83) and a control group (*n* = 83). The intervention group received a biopsychosocial integrated program, while the control group received standard psychiatric care. Assessments were conducted at baseline and 1, 2, and 3 months post-intervention using the Hamilton Rating Scale for Depression (HAMD-17), Profile of Mood States (POMS), Five Facet Mindfulness Questionnaire (FFMQ), Mental Health Continuum-Short Form (MHC-SF), and the Generic Quality of Life Inventory-74 (GQOLI-74). Patient satisfaction was evaluated at 3 months.

**Results:**

The intervention group showed significantly greater improvement in depressive symptoms (F_interaction_ = 29.003, *p* < 0.001), with a higher response rate (98.75% vs. 90.24%, *p* < 0.05). Mental health scores (POMS, FFMQ, MHC-SF) improved more in the intervention group (F_interaction_ = 143.577, 11.155, 14.658; *p* < 0.001). At 3 months, GQOLI-74 scores and patient satisfaction were also significantly higher (*p* < 0.05).

**Conclusion:**

The biopsychosocial intervention significantly improves depression, mental health, and quality of life in elderly patients with geriatric disease-related depression.

## 1 Introduction

The accelerating global population aging has led to a rising prevalence of geriatric diseases, with depression secondary to age-related chronic conditions emerging as a critical public health challenge impacting elderly well-being (Arnaud et al., 2022; Cavdar et al., 2024). According to the World Health Organization (WHO), approximately 14% of adults aged 60 years and older are living with a mental disorder, with depression being among the most common conditions. Mental disorders in this age group account for more than 10% of total disability-adjusted life years (DALYs), highlighting the heavy global burden (Petrovic and Gaggioli, 2020). Furthermore, nearly 80% of adults aged 65 years and above live with at least one chronic condition, and about 50% have two or more, substantially increasing the risk of comorbid depression (Khan et al., 2022). A recent meta-analysis estimated the global prevalence of major depression in the elderly at 13.3% (95% CI: 8.4–20.3%), with slightly higher rates in women (11.9%) than men (9.7%) (Abdoli et al., 2022). Another meta-analysis focusing on older adults with chronic diseases reported even higher rates, with pooled prevalence reaching over 60% in hospitalized or primary-care elderly patients (Ahmed et al., 2024). These findings underscore that depression secondary to chronic illness is not only frequent but also exerts profound impacts on functional status, rehabilitation outcomes, and quality of life. Importantly, recent studies emphasize a bidirectional relationship between depression and chronic diseases in older adults. Depression may arise as a consequence of conditions such as cardiovascular disease, diabetes, and stroke, while also worsening disease progression, reducing treatment adherence, and increasing mortality. Conversely, chronic diseases markedly elevate the risk of depression, creating a vicious cycle that impairs recovery and quality of life (Werneck and Stubbs, 2024; Zhou et al., 2024). This reciprocal link highlights the need for integrated approaches addressing both physical and psychological health.

Depression secondary to geriatric diseases refers to depressive states induced by prolonged illness, declining physical function, and compromised self-care capacity in elderly patients (Kupisz-Urbańska et al., 2024). These patients often become trapped in a vicious cycle of “disease-depression-functional deterioration” through the interaction between physical comorbidities and psychological disturbances, exhibiting significantly poorer mental health and quality of life compared to the general elderly population. Traditional biomedical approaches focus predominantly on symptom relief through pharmacological treatment, which may be insufficient for elderly patients who often face multiple comorbidities, functional decline, and psychosocial stressors such as loneliness, reduced social participation, and caregiver burden (Ma and Strelow, 2025). In contrast, a biopsychosocial model provides a more comprehensive framework by simultaneously addressing biological vulnerabilities, psychological distress, and social determinants of health.

Developing an integrated trinity therapeutic model addressing somatic, psychological, and social dimensions holds substantial significance for improving holistic health in patients with depression secondary to geriatric diseases. This trinity therapeutic framework, grounded in the biopsychosocial medical model, integrates multidisciplinary resources to establish a coordinated intervention system, potentially offering novel solutions for enhancing patient outcomes. Recent studies have demonstrated the promise of integrative interventions, including multidisciplinary collaboration, cognitive behavioral therapy (CBT) combined with social support, though systematic empirical research specifically evaluating biopsychosocial trinity interventions for elderly populations remains scarce (Jalali et al., 2024). In addition, prior studies have shown that mindfulness-based programs can effectively reduce depressive symptoms and improve cognitive function in older adults, while structured social engagement activities have been linked to better psychological well-being and reduced loneliness (Demarzo et al., 2015; Han, 2022). Multidisciplinary interventions—integrating medical management with psychosocial support—have also demonstrated improved treatment adherence and quality of life in geriatric populations (Werner et al., 2022). These findings support the rationale for incorporating mindfulness, social participation, and team-based care into a biopsychosocial framework for elderly patients with depression.

Breaking from traditional unidimensional medical paradigms, this study proposes a multidimensional intervention framework tailored for elderly patients under the biopsychosocial model. While previous work has acknowledged the value of integrated care, most interventions in older adults have remained limited to single domains—such as pharmacological management, stand-alone psychological therapies, or community-based activities—implemented in isolation rather than as a coordinated system. The originality of the present research lies in advancing beyond these fragmented approaches by developing a comprehensive, systematically structured program that integrates medical, psychological, and social components within one cohesive framework. Specifically, medical management in our study is dynamically tailored through both pharmacological adjustment and exercise-based rehabilitation; psychological support incorporates mindfulness practices and narrative-based life review to strengthen coping capacity; and social engagement is reinforced through intergenerational activities and structured family workshops to enhance support networks. By embedding these elements into a unified biopsychosocial model, our study provides a scalable, evidence-based approach tailored to the complex needs of elderly patients with depression secondary to chronic diseases. Through comparative analysis of mental health and functional improvements among 166 patients suffering from depression secondary to geriatric diseases, we investigate the efficacy of this trinity therapeutic model in enhancing psychological well-being and quality of life, aiming to provide evidence-based insights for optimizing comprehensive care strategies in geriatric depression management.

## 2 Methods

### 2.1 Participants

A cluster randomized controlled trial was conducted involving 166 elderly patients diagnosed with depression secondary to geriatric diseases, admitted to our hospital between January 2022 and January 2025. Participants were allocated at the ward level, with Ward A (83 patients) assigned to the intervention group and Ward B (83 patients) to the control group using a computer-generated randomization sequence via SPSS 26.0. This design ensured balanced baseline characteristics and sample sizes between groups.

Sample size estimation was performed based on the 17-item Hamilton Rating Scale for Depression (HAMD-17) as the primary outcome. Assuming a minimal clinically important difference of 3–5 points in HAMD-17 scores between groups (Cohen’s d ≈ 0.5), with *α* = 0.05 (two-tailed), *β* = 0.20 (80% power), and an effect size d = 0.5, PASS software calculated a required sample of 64 patients per group. Accounting for a cluster randomization design with an intraclass correlation coefficient of 0.02 and a design effect of 1.18, the adjusted sample size was 128 × 1.18 ≈ 151. After including a 10% attrition buffer, the final sample comprised 166 participants.

Eligibility criteria included: (1) meeting the International Classification of Diseases, 10th Revision (ICD-10) diagnostic criteria for depression; (2) age ≥ 60 years; (3) baseline HAMD-17 score ≥ 14 (indicating mild depression or above); (4) Mini-Mental State Examination (MMSE) score ≥ 24; and (5) ability to comply with interventions and provision of informed consent by patients or guardians. Exclusion criteria comprised: (1) history of schizophrenia, bipolar disorder, severe personality disorders, active suicidal ideation, or psychotic symptoms; (2) recent antipsychotic medication, electroconvulsive therapy, or experimental antidepressant treatments within 3 months; (3) severe neurological disorders (e.g., Alzheimer’s disease, vascular dementia, brain tumors); (4) organ failure with life expectancy < 6 months; or (5) alcohol or substance abuse. Attritional criteria included (1) voluntary withdrawal by participants or termination of intervention at the request of legal guardians, or (2) loss to follow-up (defined as missing two consecutive assessments or intervention sessions). During the study, 3 participants in the intervention group and 1 in the control group withdrew, resulting in 80 and 82 completers, respectively. Baseline characteristics are summarized in Table 1. The study protocol was approved by the Ethics Committee of Wenzhou Seventh People’s Hospital (approval number: EC-20210826-23).

**Table 1:** Comparison of baseline characteristics between intervention and control groups ( $\bar{X}\pm S$ )/*n* (%).

Variable	Intervention group (*n* = 80)	Control group (*n* = 82)	*χ* ^2^ */t*	*P*
Age (years)	68.67 ± 5.40	69.18 ± 6.00	−0.566	0.572
Gender			0.036	0.850
Male	32 (40.00)	34 (41.46)		
Female	48 (60.00)	48 (58.54)		
BMI (kg/m^2^)	21.01 ± 1.65	21.12 ± 1.79	−0.405	0.686
Geriatric diseases			0.015	0.902
≥ 2	49 (61.25)	51 (62.20)		
< 2	31 (38.75)	31 (37.80)		
Disease duration (months)	6.79 ± 2.04	6.55 ± 2.27	0.704	0.483
MMSE score	26.18 ± 2.16	26.02 ± 2.23	0.436	0.663
Educational level			0.498	0.780
Primary school or below	30 (37.50)	27 (32.93)		
Secondary school	29 (36.25)	30 (36.59)		
Beyond secondary school	21 (26.25)	25 (30.49)		

### 2.2 Tests administered

All assessments were independently conducted by two psychiatric attending physicians (≥ 5 years of clinical experience) blinded to group allocation. Prior to evaluation, assessors underwent consistency training to achieve an intraclass correlation coefficient (ICC) > 0.85. Case report forms anonymized group assignments using coded identifiers, and discrepancies were resolved by a third senior physician.

① HAMD-17 score. HAMD-17 scores were assessed at baseline, 1, 2, and 3 months post-intervention. This scale comprises 17 items (total score range: 0–52), with severity categorized as follows: 0–7 (no depression), 8–13 (mild depression), 14–18 (moderate depression), and ≥ 19 (severe depression) (Hassanzadeh et al., 2024).
② Clinical efficacy. At 3 months, clinical response was classified based on HAMD-17 score changes: marked improvement (≥ 50% reduction), partial improvement (20–49% reduction), or no improvement (< 20% reduction or worsening). Total response rate was calculated as (marked + partial improvements)/total participants × 100%.
③ Mental health indicators. Mental health status was evaluated using the Profile of Mood States (POMS) (López-Jiménez et al., 2021), Five Facet Mindfulness Questionnaire (FFMQ) (Shallcross et al., 2020), and Mental Health Continuum-Short Form (MHC-SF) (Plouffe et al., 2022) at baseline, 1, 2, and 3 months post-intervention. The POMS assesses seven mood domains across 40 items (total score: 0–200; lower scores indicate better mood). The FFMQ measures five mindfulness facets, including observing, describing, acting with awareness, non-judging, and non-reactivity (score range: 39–195; higher scores reflect stronger mindfulness). The MHC-SF evaluates emotional, psychological, and social well-being (score range: 14–70; higher scores denote better mental health).
④ Quality of life assessment. The Generic Quality of Life Inventory-74 (GQOLI-74) (Gao et al., 2023) was administered at baseline and 3 months after intervention, assessing four domains (physical function, psychological function, social function, and material life status), each standardized to 0–100 (higher scores indicate superior quality of life).
⑤ Satisfaction evaluation. A self-designed 5-point Likert-scale questionnaire (Cronbach’s *α* = 0.82) assessed 3-month post-intervention satisfaction across three dimensions: program utility (1–5), provider professionalism (1–5), and overall satisfaction (1–5). Total scores were categorized as very satisfied (13–15), satisfied (10–12), neutral (6–9), or dissatisfied (< 6).

### 2.3 Procedures

Both groups received interventions for 3 months.

#### 2.3.1 Control group

The control group received standard psychiatric care, which involved physician-led management following clinical guidelines for geriatric disease treatment, including routine medication prescriptions based on diagnostic results. At diagnosis, healthcare providers informed caregivers about the confirmed disease entity, clinical status, and necessary precautionary measures. Monthly mental health lectures were conducted for all patients, covering topics such as common psychological symptoms and coping strategies. Post-discharge, monthly outpatient follow-ups included blood pressure and glucose monitoring. Medication dosages were adjusted if patients reported changes in somatic symptoms.

#### 2.3.2 Intervention group

The intervention group underwent a tripartite biopsychosocial intervention program.

**Figure 1:**
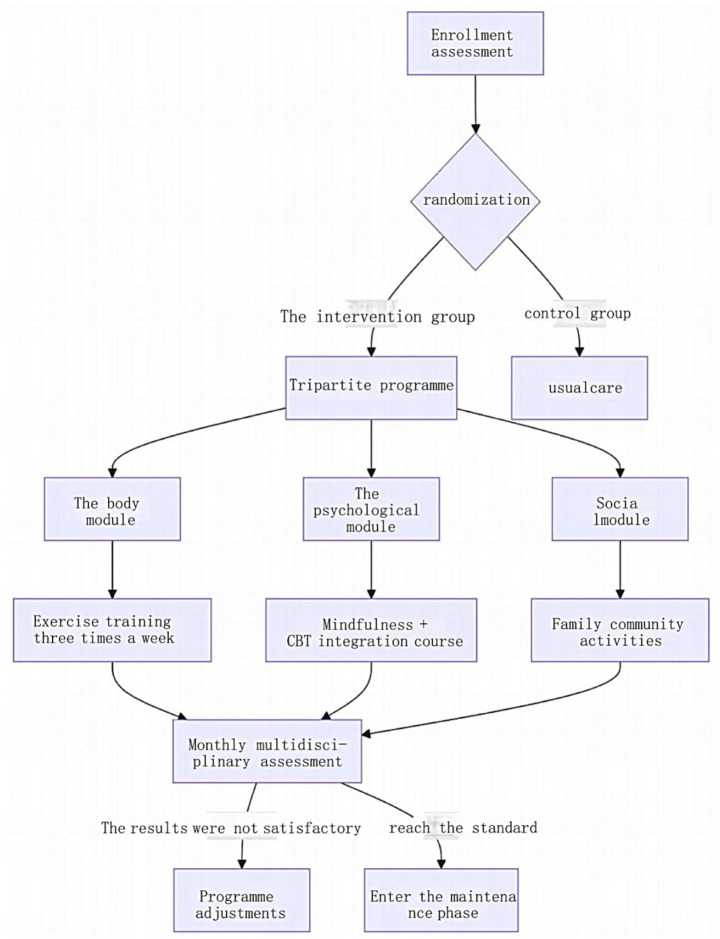
Core framework of the tripartite biopsychosocial intervention program.

1. Team composition and role allocation. A multidisciplinary team was established, comprising a medical subgroup (geriatricians, rehabilitation therapists, and nurses), a psychological subgroup (psychologists and social workers), and a social support subgroup (family coordinators and community staff). Real-time team communication was facilitated through a dedicated WeChat group with separate channels for medical, psychological, and social support. Weekly meetings were held every Wednesday to review patient progress and coordinate tasks.
2. Standardized intervention. ① Biological module: Geriatricians conducted biweekly face-to-face follow-ups to adjust pharmacotherapy based on vital signs (blood pressure, glucose, and lipid levels). Rehabilitation therapists designed tailored Baduanjin exercise plans, delivered in three 45-min supervised group sessions weekly at the rehabilitation center of our hospital. Patients were assigned daily home exercises (15–20 min of stretching or walking) monitored via smart wristbands, with data synchronized to APP for remote supervision and adjustment. Nurses distributed simplified cognitive behavioral therapy for insomnia (CBT-I) manuals, comprising sleep hygiene education, stimulus control therapy, and sleep restriction therapy, accompanied by standardized implementation protocols. Psychologists conducted weekly video consultations for sleep regulation guidance. ② Psychological module: Psychologists conducted two weekly 60-min group sessions involving mindfulness-based breathing, body scanning, and meditation. Patients completed daily 15-min APP-guided mindfulness exercises with audio prompts and progress tracking. Monthly 90-min group activities encouraged life review and shared reflections on significant experiences. ③ Social module: Community workers organized monthly intergenerational social activities (e.g., craft workshops, storytelling sessions) and facilitated patient engagement in volunteer roles (e.g., library assistance, plant maintenance) based on individual interests. Weekly 60-min family workshops educated caregivers about depression secondary to geriatric diseases, communication strategies, and home care techniques.
3. Quality control. Intervention adherence was monitored via the APP, requiring ≥ 70% completion of psychological exercises (based on literature-defined thresholds for therapeutic efficacy) (Yao et al., 2025). Initial non-compliance triggered automated reminders and phone follow-ups, and persistent non-compliance (≥ 2 consecutive weeks) prompted supplemental interventions (family supervision or task simplification). Exercise intensity was regulated using a heart rate threshold [(220 − age) × 0.6]. Patients with a ≥ 5-point increase in HAMD-17 scores during intervention were referred for further evaluation. Team performance and protocol effectiveness were reviewed monthly, with iterative adjustments to optimize outcomes. The core framework of the tripartite biopsychosocial intervention is illustrated in Figure 1.

### 2.4 Statistical procedures

All data were analyzed using SPSS version 22.0. Categorical variables, including clinical efficacy, overall satisfaction, and gender, were expressed as frequencies (percentages) and analyzed using chi-square tests. Continuous variables conforming to a normal distribution were presented as mean ± standard deviation ( $\bar{X\;}\pm S$ ). Between-group comparisons at individual time points were performed using independent samples *t*-tests, while longitudinal changes across multiple time points were assessed via repeated-measures analysis of variance (ANOVA). Figures were generated using SPSS 22.0 and GraphPad Prism 8. A *p*-value < 0.05 was considered statistically significant.

## 3 Results

### 3.1 Comparison of HAMD-17 scores

**Figure 2:**
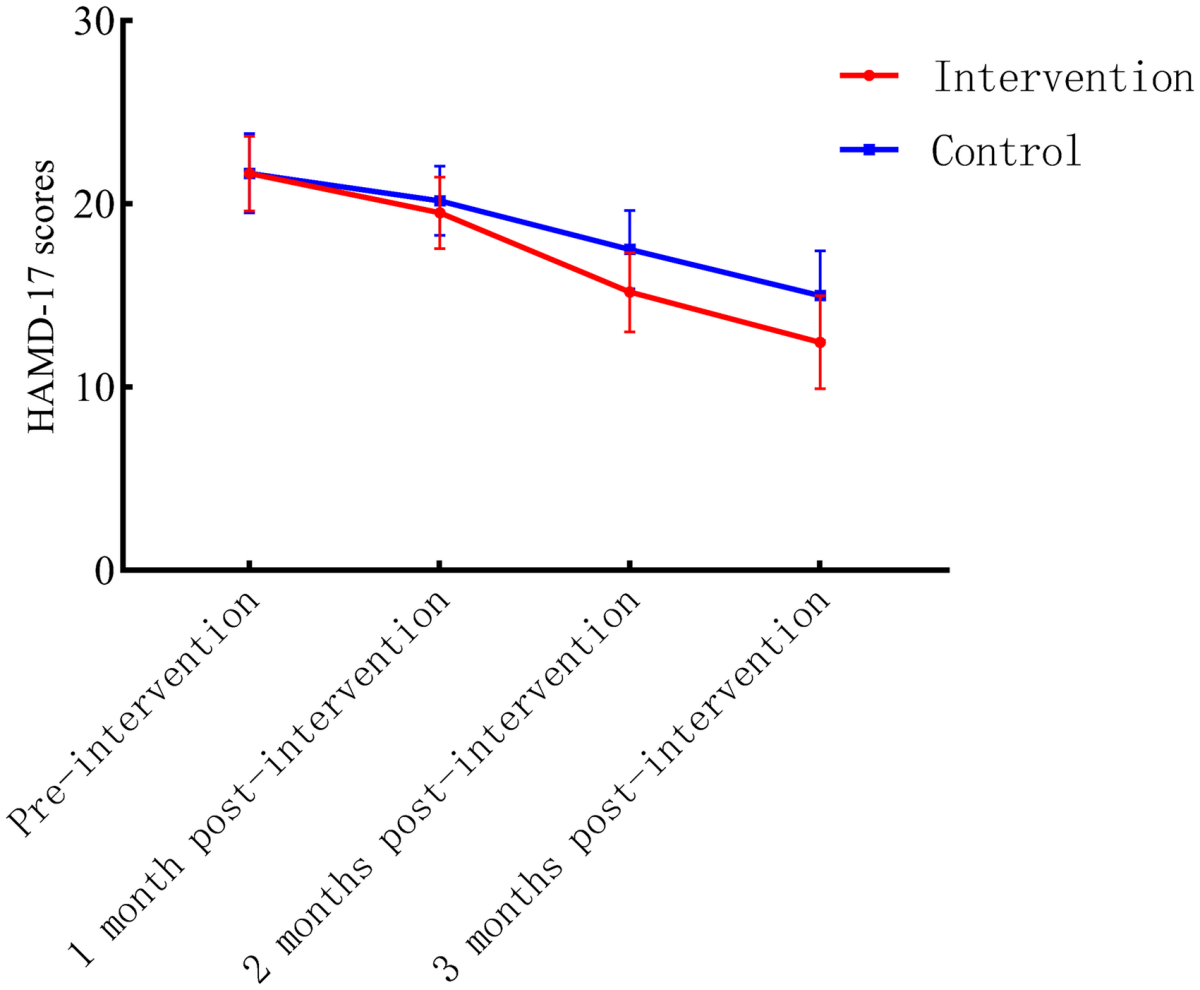
Longitudinal trends in HAMD-17 scores for intervention and control groups.

No significant differences in baseline HAMD-17 scores were observed between the intervention and control groups (*p* > 0.05). Longitudinal analysis revealed a statistically significant interaction effect of group and time (F_interaction_ = 29.003, *p* < 0.001), with the intervention group demonstrating superior improvement in depressive symptoms compared to the control group across all follow-up time points (1, 2, and 3 months; *p* < 0.05 for all between-group comparisons). Detailed scores at each assessment interval are presented in Table 2. The trajectory of HAMD-17 score changes over time is illustrated in Figure 2.

**Table 2:** Comparison of HAMD-17 scores between groups ( $\bar{X}\pm S$ ).

Group	*n*	Pre-intervention	1 month post-intervention	2 months post-intervention	3 months post-intervention	*F_time_*	*F*_group_	*F*_interaction_
Intervention	80	21.66 ± 2.04	19.51 ± 1.94	15.18 ± 2.16	12.45 ± 2.53	947.855	24.876	29.003
Control	82	21.67 ± 2.16	20.17 ± 1.89	17.50 ± 2.13	14.99 ± 2.46			
*t*		−0.025	−2.189	−6.889	−6.477	-	-	-
*P*		0.980	0.030	<0.001	<0.001	<0.001	<0.001	<0.001

### 3.2 Comparison of clinical efficacy

The intervention group demonstrated a significantly higher total response rate of 98.75% compared to 90.24% in the control group, with a statistically significant difference observed between groups (*p* = 0.043). Detailed efficacy outcomes are summarized in Table 3.

**Table 3:** Comparison of clinical efficacy between groups *n* (%).

Group	*n*	Marked improvement	Partial improvement	No improvement	Total response rate
Intervention	80	30 (37.50)	49 (61.25)	1 (1.25)	79 (98.75)
Control	82	5 (6.10)	69 (84.15)	8 (9.76)	74 (90.24)
*χ* ^2^					4.081
*P*					0.043

### 3.3 Comparison of mental health outcomes

Baseline scores for POMS, FFMQ, and MHC-SF showed no significant differences between groups (*p* > 0.05). Longitudinal analyses revealed significant group × time interaction effects for all mental health indicators (POMS: F_interaction_ = 143.577; FFMQ: F_interaction_ = 11.155; MHC-SF: F_interaction_ = 14.658; *p* < 0.001 for all), with the intervention group demonstrating superior improvements compared to the control group at each follow-up interval (1, 2, and 3 months; *p* < 0.05 for all between-group comparisons). Detailed score trajectories and intergroup differences are presented in Table 4.

**Table 4:** Comparison of mental health outcomes between groups ( $\bar{X}\pm S$ ).

Indicator	Group	*n*	Pre-intervention	1 month post-intervention	2 months post-intervention	3 months post-intervention	*F_time_*	*F*_group_	*F*_interaction_
POMS	Intervention	80	126.44 ± 10.78	101.79 ± 8.79	81.42 ± 11.81	62.84 ± 8.83	1512.641	128.805	143.577
	Control	82	125.71 ± 11.56	112.67 ± 9.29	103.76 ± 10.44	91.26 ± 11.11			
	*t*		0.416	−7.656	−12.760	−17.989	-	-	-
	*P*		0.678	<0.001	<0.001	<0.001	<0.001	<0.001	<0.001
FFMQ	Intervention	80	101.67 ± 12.18	113.56 ± 12.01	120.72 ± 8.70	127.17 ± 5.64	338.705	23.350	11.155
	Control	82	101.05 ± 11.43	105.68 ± 8.97	112.54 ± 7.66	120.85 ± 7.71			
	*t*		0.338	4.724	6.360	5.966	-	-	-
	*P*		0.736	<0.001	<0.001	<0.001	<0.001	<0.001	<0.001
MHc-sF	Intervention	80	20.30 ± 4.45	25.15 ± 6.10	28.77 ± 5.67	34.79 ± 5.71	364.017	15.033	14.658
	Control	82	20.15 ± 4.11	23.10 ± 4.35	25.88 ± 5.02	29.71 ± 5.15			
	*t*		0.228	2.461	3.445	5.953	-	-	-
	*P*		0.820	0.015	<0.001	<0.001	<0.001	<0.001	<0.001

### 3.4 Comparison of GQOL-74 scores

No significant differences in baseline GQOLI-74 domain scores were observed between the intervention and control groups (*p* > 0.05). At the 3-month follow-up, the intervention group exhibited significantly greater improvements across all domains of the GQOLI-74 compared to the control group (all *p* < 0.001), with psychological function demonstrating the most pronounced enhancement (*t* = 13.106, *p* < 0.001). Domain-specific score trajectories and intergroup differences are detailed in Table 5.

**Table 5:** Comparison of GQOLI-74 scores between groups ( $\bar{X}\pm S$ ).

Group	*n*	Physical function		Psychological function		Social function		Material life status	
		Pre-intervention	3 months post-intervention	Pre-intervention	3 months post-intervention	Pre-intervention	3 months post-intervention	Pre-intervention	3 months post-intervention
Intervention	80	69.88 ± 6.47	80.31 ± 5.76	51.49 ± 6.66	78.58 ± 6.67	65.49 ± 5.79	78.08 ± 6.09	63.16 ± 6.65	79.50 ± 8.04
Control	82	68.50 ± 5.16	76.24 ± 6.01	50.74 ± 7.06	65.73 ± 5.78	65.05 ± 6.61	73.51 ± 5.18	63.13 ± 5.84	72.50 ± 6.74
*t*		1.498	4.400	0.689	13.106	0.449	5.144	0.029	6.011
*P*		0.136	<0.001	0.492	<0.001	0.654	<0.001	0.977	<0.001

### 3.5 Comparison of patient satisfaction

**Figure 3:**
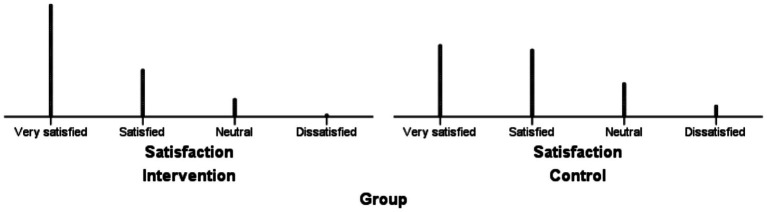
Distribution of satisfaction ratings in intervention and control groups.

The intervention group demonstrated significantly higher overall satisfaction than the control group (*Z* = −3.141, *p* = 0.002), with 62.50% of intervened participants reporting “very satisfied” compared to 39.02% in the control group. Categorical distributions of satisfaction ratings across both groups are detailed in Table 6, while Figure 3 visually summarizes these contrasts.

**Table 6:** Comparison of patient satisfaction between groups *n* (%).

Group	*n*	Very satisfied	Satisfied	Neutral	Dissatisfied
Intervention	80	50 (62.50)	21 (26.25)	8 (10.00)	1 (1.25)
Control	82	32 (39.02)	30 (36.59)	15 (18.29)	5 (6.10)
*Z*		−3.141			
*P*		0.002			

## 4 Discussion

The accelerating global aging population has precipitated a surge in geriatric diseases, with depression secondary to these conditions emerging as a critical public health challenge (Pijnenburg et al., 2024; Kovacheva et al., 2025). Naguy and Al-Khadhari (2024) Estimate that approximately 40% of elderly patients with chronic illnesses experience comorbid depressive symptoms. Miletic et al. (2025) have further emphasized that depressive symptoms profoundly impede rehabilitation outcomes in elderly patients, compromising their self-care capacity and diminishing social engagement. Conventional biomedical approaches, predominantly focused on somatic symptom management, often neglect the psychosocial dimensions of care, thereby failing to address the multifaceted health needs of this vulnerable population. Therefore, developing effective interventions holds significant implications for improving mental health and quality of life in elderly patients with secondary depression. In this context, the tripartite biopsychosocial intervention program developed in this study represents a paradigm shift, innovatively synergizing somatic, psychological, and social interventions to holistically alleviate depressive symptoms and enhance overall quality of life in elderly patients with depression secondary to geriatric diseases, suggesting promising applicability in geriatric mental health management.

At the level of somatic intervention, the program achieved precise and systematic management of physical symptoms through dynamic medication adjustment, Baduanjin training, and sleep management. Patients in the intervention group showed a reduction in HAMD-17 scores over three months, significantly more obvious than the decrease in the control group. This may be attributed to the program’s multidimensional somatic intervention. Regular Baduanjin training improves neuroplasticity by promoting the secretion of brain-derived neurotrophic factor (BDNF), and real-time monitoring of exercise data via smart bracelets increases patient compliance, effectively regulating the hypothalamic–pituitary–adrenal (HPA) axis and reducing cortisol levels, which aligns with emerging evidence on exercise-induced neuroplasticity. For instance, Zhao et al. (2024) demonstrated that mind–body exercises like Baduanjin enhance HPA axis regulation and promote neurotrophic factor secretion, which are critical for mood stabilization. Combined with CBT-I and sleep monitoring, sleep efficiency in the intervention group improved significantly, further alleviating depressive symptoms. Similarly, a meta-analysis by Marinelli et al. (2024) highlighted the additive benefits of combining physical activity with psychological interventions. Moreover, it has been demonstrated that regular exercise regimens can not only enhance physical conditioning but also optimize sleep architecture, thereby facilitating improved engagement in daily activities and elevating quality of life within the physical functional domain (Hossain et al., 2024). In contrast to conventional care relying solely on medication adjustment, the program’s collaboration among geriatricians, rehabilitation therapists, and nurses enables dynamic tracking of physical indicators such as blood pressure and blood glucose. For example, heart rate variability monitoring was used to adjust antihypertensive medication for patients with hypertension, highlighting the advantages of multidisciplinary cooperation in somatic management.

Psychological improvement potentially stems from the deep integration of mindfulness training and cognitive restructuring. The intervention group significantly enhanced mindfulness levels (remarkably increased FFMQ scores), mood states (significantly reduced POMS scores), and psychological well-being (obviously enhanced MHc-sF scores) through twice-weekly group mindfulness sessions, daily APP-guided practices, and monthly life review activities. The core mechanisms may be as follows: mindfulness meditation enhances prefrontal cortex regulation of the amygdala, reducing excessive responses to negative emotions (Kral et al., 2018). Van der Velden et al. (2023) have also shown that a clinically effective mindfulness intervention modulates neurocognitive functioning during depressive rumination and the ability to sustain attention to the body. As a form of narrative therapy, life review helps patients reconstruct self-worth, consistent with the significant improvement in psychological function. In line with a mixed method study by Kwan et al. (2019), the nurse-led short-term life-review intervention significantly enhanced spiritual well-being, with participants endorsing the intervention as both acceptable and clinically meaningful. Compared with traditional individual psychotherapy, the program’s gamified APP design (e.g., point rewards, progress tracking) boosts mindfulness practice compliance, demonstrating how technology enhances the accessibility and sustainability of psychological intervention.

As for social function, its enhancement relies on the dual construction of community resource integration and family support systems. Through monthly intergenerational activities (e.g., intergenerational handicraft making), personalized community volunteer services (e.g., library organization, plant care), and weekly family workshops, the intervention group’s social function score increased, significantly higher than the control group. The underlying mechanisms may be related to that community activities enhance social engagement through “intergenerational reciprocity,” increasing weekly social interaction and reducing loneliness. Family workshops improve caregivers’ empathy and communication skills, and reduce family conflicts, creating a more supportive family environment. Our findings resonate with Liu et al. (2024) assertion that robust social support networks buffer against anxiety and depression in older adults, while Li et al. (2024) emphasized the role of community continuity in sustaining mental health gains. In contrast to the “fragmented” support of single mental health lectures in conventional care, the program’s precise intervention by community workers, through “needs assessment-resource matching,” customizes social participation plans for patients (e.g., assisting painting-talented patients in community art gallery volunteering), increasing social function recovery rates. This demonstrates the unique advantages of the “hospital-community-family” linkage model in social support.

Compared with traditional interventions, the core innovation of our program lies in breaking through single-discipline limitations. Through real-time communication among medical, psychological, and social support teams (e.g., specialized WeChat groups, weekly meetings) and technology empowerment (smart device data synchronization, APP intervention quality control), it achieves integrated intervention from somatic symptom control to comprehensive psychological and social function rehabilitation. This study showed that the intervention group’s total effective rate was 98.75%, significantly higher than 90.24% in the control group, with the improvement in psychological and social functions far exceeding conventional care. This synergistic effect of “precise somatic management-deep psychological intervention-extensive social support” not only relieves depressive symptoms but also enhances overall quality of life through multiple pathways of neural remodeling, cognitive restructuring, and social capital reconstruction, providing a scalable practice paradigm for the comprehensive intervention of depression secondary to geriatric diseases.

Despite these promising results, several limitations warrant consideration. First, the single-center design and moderate sample size may constrain the generalizability of findings. Second, the 3-month intervention period precludes assessment of long-term efficacy, particularly regarding relapse prevention. Third, while the biopsychosocial model demonstrated superiority over conventional care, the relative contributions of individual intervention components (e.g., Baduanjin vs. mindfulness training) remain to be disentangled. Future multicenter trials with extended follow-up periods and factorial designs are needed to optimize intervention protocols and validate scalability.

## 5 Conclusion

This cluster randomized controlled trial provides robust empirical support for the biopsychosocial integrated intervention as a transformative approach to managing depression secondary to geriatric diseases. By concurrently addressing biological vulnerabilities, cognitive-emotional distortions, and social determinants of health, this model transcends the reductionist limitations of traditional care paradigms. As global healthcare systems grapple with the complexities of aging populations, such integrative frameworks offer a viable pathway to enhancing both clinical outcomes and patient-centered care quality.

## Data Availability

The original contributions presented in the study are included in the article/supplementary material, further inquiries can be directed to the corresponding author.
